# Identification of an *mcr*-9-Carrying Salmonella enterica Serotype Heidelberg Strain Isolated from Blood

**DOI:** 10.1128/MRA.00676-21

**Published:** 2021-08-26

**Authors:** Khansaa Abdullah, Peter C. Iwen, Baha Abdalhamid

**Affiliations:** a Department of Pathology and Microbiology, University of Nebraska Medical Center, Omaha, Nebraska, USA; b Nebraska Public Health Laboratory, University of Nebraska Medical Center, Omaha, Nebraska, USA; University of Maryland School of Medicine

## Abstract

Salmonella enterica, represented by a large number of serotypes, can cause a broad variety of diseases that range from mild gastroenteritis to severe systemic infections. This report provides a draft genome sequence of an *mcr*-9-carrying Salmonella enterica  serotype Heidelberg strain isolated from blood.

## ANNOUNCEMENT

Salmonella enterica has been associated with foodborne gastroenteritis worldwide, which in rare instances may lead to an invasive infection, especially in the immunocompromised, the elderly, and young children (1, 2). Invasive salmonellosis diseases usually require antibiotic treatment. Antimicrobial agents such as ciprofloxacin or ceftriaxone are considered standard therapy; however, in cases where Salmonella is highly drug resistant, colistin may be regarded as a last-line antibiotic for treatment (2, 3). Despite being deemed rare for the Gram-negative bacilli, *mcr*-like genes have been recognized worldwide as the genes responsible for colistin resistance in the last few years (2, 3). We present in this report the draft genome sequences of an *mcr*-9-carrying Salmonella enterica serotype Heidelberg strain isolated from the blood of a patient. The sample was deidentified; therefore, the study did not require approval from an institutional review board (IRB).

A positive blood culture bottle (Bactec 9240; Becton, Dickinson, San Diego, CA) was cultured on blood, MacConkey, and chocolate agar plates and incubated overnight at 5% CO_2_ and 37°C. The MicroScan WalkAway system (Beckman Coulter, CA) was used for organism identification and antimicrobial susceptibility testing. After overnight culture under the same conditions as used for isolation, genomic DNA extraction was performed on a single colony using the MagNA Pure compact nucleic acid isolation kit I (Roche Diagnostics, IN, USA). A NanoDrop 2000 UV-visible (UV-Vis) spectrophotometer (Thermo Fisher, MA, USA) and a Qubit 3.0 fluorometer (Invitrogen, CA, USA) were used for DNA concentration and quality detection, respectively. DNA libraries were constructed from the extracted bacterial genomic DNA using the Nextera XT library prep kit (Illumina, CA, USA). Using 2 × 250-bp paired-end chemistry, the MiSeq platform (Illumina) was used for whole-genome sequencing. Quality assessment of the run was determined using the following parameters: Phred quality score, QS30 > 75%; clusters passing filters, >80%; and cluster density, 600 to 1,300. FastQC v0.11 and Trimmomatic v0.33 were used for FASTQ quality read and trimming, respectively, while SPAdes v3.15 and BBMap v38.84 were used for *de novo* assembly and fasta filtration, respectively (4–7). QUAST v4.1 was used for quality checking of the assembly (8). The NCBI Prokaryotic Genome Annotation Pipeline (PGAP) v4.8 was used for genome annotation, while MLST v2.0 was used for multilocus sequencing typing (9, 10). Using SeqSero v1.2 software, the organism serotype was determined to be Heidelberg (11). Resistance genes were detected using ABRicate v1.0 with the CARD database (12, 13). Unless otherwise specified, default parameters were used for all software.

The total length of the genome sequence was 5,301,098 bp, with a GC content of 51.93%. The total number of contigs was 143; the largest contig was 773,925 bp, and the *N*_50_ value was 240,044 bp. The total number of reads was 676,363, and the coverage depth was 67.64×.

Interestingly, this Salmonella serotype carried an *mcr*-9 gene; reports in the literature indicate that *mcr*-9-carrying Salmonella strains frequently are not associated with colistin resistance in the United States (14).

### Data availability.

This isolate is available at https://www.ncbi.nlm.nih.gov/ under the BioProject accession number PRJNA230403, the BioSample accession number SAMN17813371, and the Sequence Read Archive (SRA) accession number SRS8205147.
